# Adalimumab (TNF**α** Inhibitor) Therapy Exacerbates IgA Glomerulonephritis Acute Renal Injury and Induces Lupus Autoantibodies in a Psoriasis Patient 

**DOI:** 10.1155/2013/812781

**Published:** 2013-07-24

**Authors:** S. S. Wei, R. Sinniah

**Affiliations:** ^1^Kidney and Hypertension Clinic, St John of God Healthcare, Subiaco, WA, Australia; ^2^Department of General Medicine, Sir Charles Gairdner Hospital, Nedlands, WA, Australia; ^3^Department of Pathology, Royal Perth Hospital, Perth, WA, Australia

## Abstract

Adalimumab (Humira) is a tumour necrosis factor **α** (TNF**α**) inhibitor that is approved for the treatment of rheumatoid arthritis, psoriasis, psoriatic arthritis, Crohn's disease, ankylosing spondylitis, and juvenile idiopathic arthritis (Sullivan and Preda (2009), Klinkhoff (2004), and Medicare Australia). Use of TNF**α** inhibitors is associated with the induction of autoimmunity (systemic lupus erythematosus, vasculitis, and sarcoidosis or sarcoid-like granulomas) (Ramos-Casals et al. (2010)). We report a patient with extensive psoriasis presenting with renal failure and seropositive lupus markers without classical lupus nephritis after 18 months treatment with adalimumab. He has renal biopsy proven IgA nephritis instead. Renal biopsy is the key diagnostic tool in patients presenting with adalimumab induced nephritis and renal failure. He made a remarkable recovery after adalimumab cessation and steroid treatment. To our knowledge, this is a unique case of a psoriasis patient presenting with seropositive lupus markers without classical lupus nephritis renal failure and had renal biopsy proven IgA glomerulonephritis after receiving adalimumab.

## 1. Introduction

A 61-year-old man was referred for evaluation of his compromised renal function. He has had chronic extensive plaque psoriasis since 2002 with a psoriasis area and severity index (PASI) score of 27 [1, 2]. For the past few years, he received various treatments including phototherapy, acitretin, methotrexate, cyclosporine, sulphasalazine, and leflunomide without much success until he received adalimumab 18 months prior to his first presentation to the renal clinic in 2011. There was no past history of renal disease and his renal function tests were normal (in 2009, serum creatinine level was 83 *μ*mol/L and urea was 7.2 mmol/L). His urine microscopy was normal with no proteinuria detected prior to his adalimumab treatment. He self-injected adalimumab 40 mgm fortnightly. He noted transient injection site redness that lasted for a few days after each injection. When routine blood tests showed that he had renal failure with serum creatinine level of 282 *μ*mol/L, urea of 20.3 mmol/L, and estimated glomerular filtration rate (eGFR) of 21 mL/min/1.73 m² calculated by the abbreviated MDRD equation [3], his attending dermatologist referred him to the renal clinic for further management. On presentation, he did not have any urinary symptoms such as haematuria or dysuria. However, he had a few weeks history of feeling tired with some appetite loss. He had no skin rash, fever, or new joint pain.

He developed hypertension in 2008 but was only started on antihypertensive medication, perindopril, in 2009. His blood pressure control has been poor after he received adalimumab treatment. He is also on venlafaxine (Efexor) for his depression. He denied intake of NSAIDs. He worked as a tiler. He takes wine socially and he had 25 pack-years of smoking before quitting 10 years ago. There was no history of allergy.

On clinical examination, the patient was pale and afebrile. Blood pressure was 180/80 mmHg. Heart and lungs were clear and there were no vasculitic spots noted. His abdomen was soft and the liver was palpable 2 cm below costal margin. Kidneys were not palpable. No obvious renal bruits were heard. There was no obvious psoriasis plaques or joints deformity noted. 

Initial laboratory tests showed increased serum creatinine level of 285 *μ*mol/L, urea of 20.6 mmol/L, and normal electrolytes. Urine microscopy showed RBC > 100, WBC < 10, and protein 2+. No red cell casts were noted. Urine protein creatinine ratio (urine PCR) was elevated at 0.135 g/mmol (*N* < 0.015). Urine albumin creatinine ratio (urine ACR) was 74.7 mgm/mmol (*N* < 2.5). Liver function was normal except that GGT was 90 U/L. He had anaemia with Hb of 106 gm/L and platelets were 263 (Hb was 141 gm/L in 2008). CRP was normal and uric acid was 0.53 mmol/L. Calcium and phosphate levels were normal. ANA was positive at 1 : 320 and anti-dsDNA was positive at 21 IU/mL (*N* < 4.2). RA was negative. ANCA was negative with normal MPO and PR3. Serum immunoglobulin level was not measured. The ultrasound report of the kidneys showed no hydronephrosis. The right kidney measured 119 mm in length and the left was 109 mm. The prostate was mildly enlarged at 32 mL. There was good bladder emptying.

**Figure 1:**
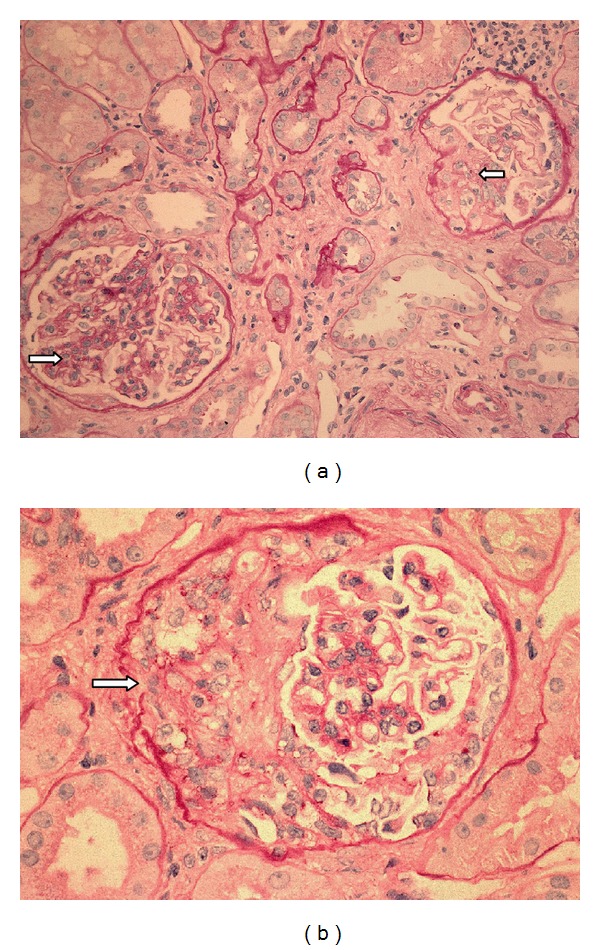
(a) The section shows glomeruli with moderate mesangial hypercellularity and a fibrocellular crescent. There is also mild tubular atrophy with tubular basement membrane thickening (PAS stain ×20). (b) Higher power shows the fibrocellular crescent with focal rupture of Bowman capsule (PAS stain ×40).

**Figure 2:**
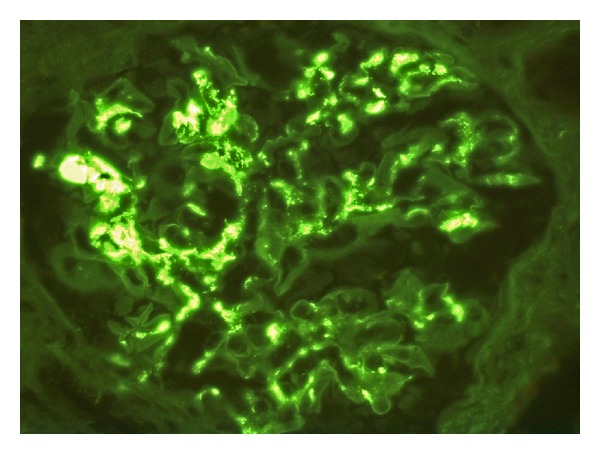
The immunofluorescence microscopy shows IgA deposits in the glomerular mesangium (magnification ×40).

Renal biopsy was performed and the biopsy specimen contained a strip of cortex and medulla with 13 glomeruli; all showed moderate mesangial hypercellularity. 8 glomeruli showed segmental sclerosis, and 8 also showed crescents, both cellular and fibrocellular with adhesion to Bowman capsule (Figures 1(a) and 1(b)). There was moderate arteriosclerosis but no vasculitis. Immunofluorescence microscopy on the renal tissue with 17 glomeruli was performed by standard techniques staining with antibodies to IgA, IgG, and IgM, complements C3c, C4c, and C1q, fibrinogen, and kappa and lambda light chains. There was positive mesangial staining for IgA (Figure 2) and complement C3c and both kappa and lambda light chains. No other immunoglobulin or C1q deposits were present. The diagnosis was IgA mesangioproliferative glomerulonephritis with 61.5% segmental glomerulosclerosis and crescents, mild tubular atrophy and interstitial fibrosis (20% involvement), and moderate arteriosclerosis.

His adalimumab was ceased and prednisolone was started at a dose of 1 mgm/kg body weight. The prednisolone dosage was gradually reduced by 10 mgm per week when his renal function showed improvement. His blood pressure reading remained high at 160/90 and it was brought under control with amlodipine and candesartan HCT. His renal function started to show improvement 3 weeks later with a return of near normal serum creatinine level of 112 *μ*mol/L and urea of 11.0 mmol/L at 20th month follow-up. His urine PCR rose to 0.520 g/mmol (*N* < 0.015) with urine ACR level of 190 mgm/mmol (*N* < 2.5) before returning to normal level at the 9th month. Urine microscopy also returned to normal at the 12th month. His Hb improved to 136 gm/L. Anti-dsDNA still remained positive at 5.9 IU/mL (*N* < 4.2) and ANA was reduced to 1 : 40. 

## 2. Discussion

Psoriasis is a chronic disorder characterised by erythematous plaques, patches, and papules which may be pruritic and classically have silver scale. Morphologically, there are varying forms with 80–90% being of the plaque variety. Severe psoriasis involves large areas of the skin surface. Due to the chronic and unique visual nature of this disease, there can be profound psychosocial consequences [4]. Our patient's chronic and extensive plaque psoriasis failed to respond to the standard therapies like acitretin, methotrexate, cyclosporine and phototherapy instead it was brought under control with Adalimumab.

Tumour necrosis factor alpha (TNF*α*) is a major proinflammatory cytokine in many chronic inflammatory diseases, including psoriasis [5]. Anti-TNF*α* drugs are an established treatment in the management of severe psoriasis [6]. Adalimumab is a fully humanized monoclonal anti-TNF*α* antibody that binds both soluble and membrane bound TNF*α*. It is an effective targeted treatment and has been well tolerated by most patients. The common treatment side effects are headaches, injection site reaction, and diarrhea [7]. Anti-TNF*α* drugs have been linked to systemic vasculitis [8–10], although renal involvement was rare [8, 11–14]. 

To our knowledge, this is a unique case of a psoriasis patient presenting with renal failure and had renal biopsy proven IgA glomerulonephritis while on adalimumab. This patient showed the classic immunohistological features of IgA glomerulonephritis with C3c activation, the absence of other immunoglobulins, and also absence of C1q. This was the most common pattern as was seen in a large series of 239 [15] IgA nephropathy patients, in whom IgA and C3c only were found in 45.7% of the patients. In classical lupus nephritis IgA and/or IgM deposits may be found but the predominant immunoglobulin was IgG and C3c with C1q activation [16]. These features were not seen in this case despite the clinical findings of lupus antibodies. Therefore, the present case did not support classical lupus nephritis. IgA nephritis may have existed before the anti-TNF*α* treatment and was reactivated after the treatment. This is unlikely as he has normal urine and renal profile prior to the treatment.

The pathogenic role of anti-TNF*α* treatment in the development of IgA glomerulonephritis in this case is suggested by (i) the improvement in clinical and laboratory abnormalities after drug withdrawal and initiation of corticosteroid therapy and (ii) the temporal relation of new onset glomerular disease to drug use with long standing psoriasis of many years and no prior renal disease. Our patient's IgA nephropathy is not consistent with other reported psoriasis associated IgA nephropathies. Usually, when psoriasis is poorly controlled, IgA nephropathy can occur [17]. However, this case is unique in that the patient's psoriasis was well controlled, but yet he presented with severe aggressive features of a crescentic glomerulonephritis associated with IgA and C3c. 

The mechanisms involved in putative anti-TNF*α* induced IgA nephritis in psoriasis are uncertain. Guillevin and Mouthon [18] suggested that anti-TNF*α* drugs may form immune complexes, activate complement, and mediate inflammation by switching from T helper type 1 to type 2 cytokine response, thus upregulating antibody production. It is accepted that prolonged treatment with anti-TNF*α* therapy induces autoantibodies, including antinuclear antibody, antidouble-stranded DNA, and anticardiolipin antibodies, although usually these remain clinically silent and disappear after discontinuation of therapy [19]. The development of autoantibodies was acknowledged in the Safety Trial of Adalimumab in Rheumatoid Arthritis [20]; however, the data on adalimumab in psoriasis remained scarce.

This case illustrates that renal biopsy is essential in the clinical diagnosis of renal failure in the presence of seropositive markers for autoimmunity in adalimumab treated patients. IgA nephropathy can be a possibility even in the presence of nephritis and positive lupus immune markers. Baseline testing of ANA and anti-dsDNA in all psoriasis patients prior to commencing TNF*α* inhibitors therapy are recommended. The short term outcome of his IgA nephritis appears good, but the longer term prognosis is guarded as the biopsy shows a focal sclerosing and crescentic glomerulonephritis. There is a need for regular renal function tests and blood pressure checks in patients undergoing TNF*α* inhibitors therapy.
